# Integrated strategy for multivariate quality description of *Lonicera japonica* by UHPLC-HRMS approach

**DOI:** 10.1016/j.fochx.2026.104279

**Published:** 2026-08-11

**Authors:** Yumei Wang, Meiling Gu, Honglian Zhang, Yujian Han, Ruoxin Li, Yanjie Dai, Yabin Zhao, Qi Liu

**Affiliations:** a Laboratory of Serum Pharmacochemistry, Qiqihar Medical University, Qiqihar 161006, China; b School of Pharmacy, Qiqihar Medical University, Qiqihar 161006, China; c Experimental Center of Natural Medicinal Chemistry, Qiqihar Medical University, Qiqihar 161006, China

**Keywords:** Chemical indicators, UHPLC-HRMS, *Lonicera japonica*, Anti-inflammatory, Quality description

## Abstract

An overall quality description strategy was established for *Lonicera japonica* using ultra-high performance liquid chromatography combined with high-resolution mass spectrometry approach. Global chemical profiles were unscrambled *via* gradient-solvent extraction combined with information dependent acquisition, primary components were fully extracted. Quality indicators were located in view of constituents absorbed into blood, transformation inference and anti-inflammatory evaluation. The accumulated contents and correlation coefficient of quality indicators based on multiple reaction monitoring were applied for quality control. As a result, 115 of chemical profiles were identified. The contents of flavonoids, prenol lipids, alcohols and polyols were 110.00 mg/g, 58.44 mg/g, and 41.47 mg/g under the unified technology. 17 of bioactive chemical indicators were screened out. The chemical indicators' accumulations of *Lonicera japonica* in different origins ranged from 43.68 mg/g to 56.80 mg/g, and the correlation coefficient was higher than 0.85. Our study provides a novel pattern of systematic quality control for *Lonicera japonica*.

## Graphical abstract

**Figure:**
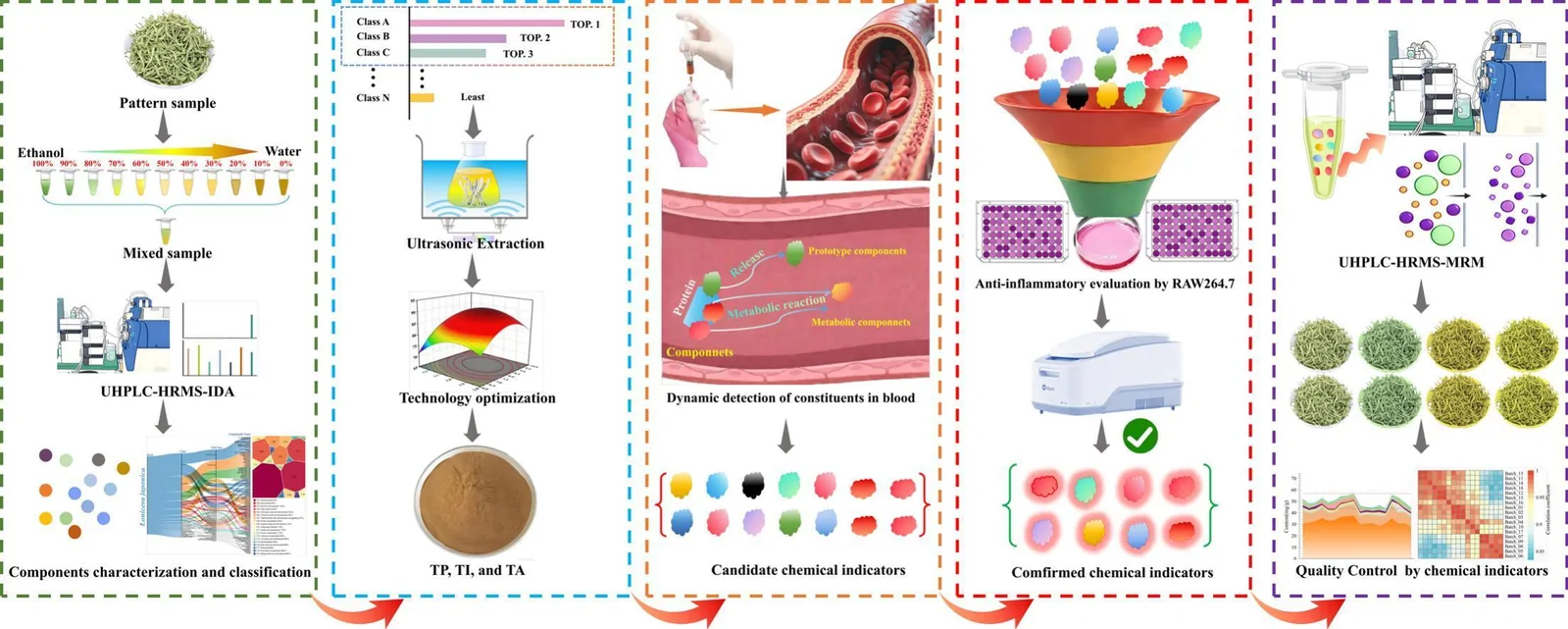

## 1 Introduction

*Lonicera japonica*, the dried flower bud as well as known as Jinyinhua in China, is a kind of famous functional food which possesses various bioactivity properties, including anti-inflammatory (Gao et al., 2024), antioxidant (Lin et al., 2024), and anticancer (Zhang et al., 2025). For the past few years, *Lonicera japonica* has been broadly used in multiple fields covered healthcare (Mei et al., 2025), food (Cao et al., 2022), cosmetics (Michalak, 2023), and feed additives (Tang, Liu, Zhong, & Fang, 2021). However, due to the increasing demand of *Lonicera japonica* in market, the wild resource are largely insufficient. Therefore, in recent years, in order to satisfy the large quantity demand of *Lonicera japonica* resource, cultivated *Lonicera japonica* was planted in various areas of China (Wang et al., 2023). Nevertheless, due to the diverse environment of planting areas, the accumulation of chemical ingredients maybe disparate, leading to the quality of *Lonicera japonica* different (He et al., 2024). Hence, comprehensive quality control system is especially vital for cultivated *Lonicera japonica*.

Previously, as for the quality control of *Lonicera japonica*, high-level components included chlorogenic acid, isochlorogenic acid A, isochlorogenic acid C and luteolin-7-O-glucoside were usually used (Wu et al., 2025). However, the phytochemical profiles of *Lonicera japonica* are diversiform, covered prenol lipids, flavonoids, terpenoids and other components (Tong et al., 2025; Zheng, Wang, Chi, & Wang, 2024), merely selecting several substances with high-content could not reflect the overall quality. Therefore, all-sided chemical indicators are more scientific for unscrambling the quality of *Lonicera japonica*.

Ultra-high performance liquid chromatography combined with high-resolution mass spectrometry (UHPLC-HRMS) is a mighty technology (Yu, Yao, & Guo, 2021), which shows great advantages in chemical understanding combined multiple analytical tools such as MSDIAL (Wartmann, Boxler, Kraemer, & Steuer, 2024), MZmine (Heuckeroth et al., 2024), and GNPS (Sheng, Wang, Liu, & Jiang, 2024). Recent years, UHPLC-HRMS has been widely applied in chemical characterization *in vitro* (Deng et al., 2026; Li et al., 2026; Shi et al., 2022; Zhang et al., 2022;) and *in vivo* (Lang et al., 2023; Zeng et al., 2020). There are different data acquisition modes like information dependent acquisition (IDA) (Zhang, He, Zeng, & Chen, 2023) and multiple reaction monitoring (MRM) (Verri Hernandes & Warth, 2024) of UHPLC-HRMS. Under IDA mode, MS and MS/MS information could be obtained, and it shows evident high-resolution advantage in identification process (Kang et al., 2020; Peng, Ge, Shang, Liu, & Jiang, 2024; Wu, Shangguan, Huang, & Wang, 2024). Besides, as for MRM acquisition, MS/MS quantitative information could be gained after eliminating the interference from large irrelevant ion, and it exhibits remarkable high-sensitivity characteristic in quantitative study (Julien, Timothée, Estelle, Aurélien, & Marc, 2022; Lin et al., 2025). Hence, the combination of IDA and MRM may simultaneously take advantages of the two technologies (Huang et al., 2023), which may exhibited powerful superiority in study of chemical indicators.

In the present paper, we first revealed the full-scale chemical profiles of *Lonicera japonica* based on distinctive gradient-solvent extraction *via* advanced UHPLC-HRMS approach under IDA mode. Then, the extract of alternative components was obtained by efficient ultrasonic-assisted technology. Then the nominated chemical indicators were located out through the overall consideration of ingredients absorbed into blood and transformation analysis of precursor ingredients. Next, the final chemical indicators were confirmed by classic anti-inflammatory activity in RAW264.7 cells induced by lipopolysaccharide (LPS). Finally, under multiple reaction monitoring (MRM) acquisition, accumulated contents and correlation coefficient of chemical indicators were adopted for the quality description of *Lonicera japonica* from different origins.

## 2 Experimental

### 2.1 Reagents, materials and animals

Acetonitrile of high performance liquid chromatography (HPLC) grade and LPS were offered by Merck Company (Darmstadt, Germany). Foline-phenol was purchased from Biotopped Life Sciences (Beijing, China). Reference substances covered rutin, gallic acid and swertiamarin were provided by Weikeqi Biotechnology (Chengdu, China). RAW264.7 cells and Dulbecco's Modified Eagle Medium (DMEM) were offered by Suzhou Haixing Biotechnology Company (Suzhou, China). Cell Counting Kit-8 (CCK-8) kit and Nitric Oxide (NO) kit were gained from Suzhou Xinsaimei Biotechnology Company (Suzhou, China) and Beyotime Biotechnology Company (Shanghai, China), respectively. Other regents such as ethanol, sodium nitrite, aluminium nitrate, sodium hydroxide, natrium carbonicum, vanillic aldehyde, glacial acetic acid and perchloric acid were of analytical grades.

18 batches of *Lonicera japonica* were obtained from different origins in China, among them, batch 1–6, batch 7–12 and batch 13–18 were collected from Shandong province, Henan province, and Hebei province, severally.

Male Sprague-Dawley rats (6–8 weeks old, weighting 200 ± 20 g) were offered by the experimental animal center in Qiqihar Medical University. The animal experiments were complied with protocols from Animal Experimental Ethics Committee of Qiqihar Medical University (Number: QMU-AECC-2021-217) and national regulations (China's “Regulations on the Management of Experimental Animals” and ARRIVE). Besides, during the animal study, experiments were in strict accordance with the institutional animal care and use policies, ensuring the animals gained humane treatment and minimization of suffering.

### 2.2 Chemical recognition of *Lonicera japonica*

#### 2.2.1 Sample preparation

18 batches of *Lonicera japonica* were crushed and filtered through a 3-mesh sieve, then equal weight of *Lonicera japonica* powder was mixed and a pattern sample was obtained. The extraction procedures were designed according to our characteristic method (Wang et al., 2025): Totally 11 of *Lonicera japonica* powder weighting 0.5 g were severally taken out and extracting for 30 min by ultrasonic with 10 mL of gradient-solvent which covered different proportions of ethanol (water, 10% ethanol, 20% ethanol, 30% ethanol, 40% ethanol, 50% ethanol, 60% ethanol, 70% ethanol, 80% ethanol, 90% ethanol, absolute ethyl alcohol). Then the solution was centrifuged at 12000 r/min for 5 min and the supernatant was separated. Finally, after mixing with equal volume and filtering by a membrane, 11 of samples were received and further analyzed through UHPLC-HRMS under IDA mode.

#### 2.2.2 UHPLC-HRMS conditions under IDA mode

Chromatographic parameters: An ultra-efficient C18 column (1.8 μm, 2.1 × 100 mm) was used and the temperature was set at 40 °C. Acetonitrile and water both consisted of 0.1% formic acid were employed as organic phase (A) and aqueous phase (B) severally. The elution program was as follows: 0–2.5 min, 5–25% A; 2.5–3.5 min, 25–50% A; 3.5–7.5 min, 50–75% A; 7.5–8 min, 75–100% A, 8–12 min, 100% A. The injection volume was maintained at 5 μL. Under these conditions, the effluent was further acquired by mass spectrometry.

Mass spectrometry methods: The temperature of electrospray ionization source was 500 °C under curtain gas of 35 L/min, nebulizing gas of 55 L/min and auxiliary gas of 55 L/min. Mass during 50–1500 Da was collected under 5500 V in positive ion mode (POS) and − 4000 V in negative ion mode (NEG). Primary mass data was obtained under declustering potential (DP) of 100 V and collision energy (CE) of 10 V. MS/MS data was acquired by IDA mode, and DP was set at 100 V, CE was during 20–60 V.

#### 2.2.3 Chemical identification

Firstly, through searching various databases like China National Knowledge Infrastructure, Wanfang, Pubmed, Web of Science, and Sci Finder, a chemical database covered comprehensive information was customized beforehand. Then the acquired data file was imported into the MSDIAL software, after setting the corresponding loading forms (POS: + H, + NH_4_, + H −H_2_O; NEG: − H, + HCOO), alternative ions were obtained. Subsequently, in view of the fragmentation pattern, mass spectrometry analyzing experience, self-building database, online database and MS/MS database, the components in *Lonicera japonica* are finally identified.

### 2.3 Extraction optimization of flavonoids, prenol lipids, alcohols and polyols

#### 2.3.1 Standard curves establishing

UV–visible spectrum was employed to establish the standard curves of rutin, swertiamarin and gallic acid. Then the yields of total flavonoids (TF), total prenol lipids (TP), total alcohols and polyols (TA) in *Lonicera japonica* were determined.

Rutin curve was established according to the classic method (Wang et al., 2025): Different volumes of rutin solution (0.2092 mg/mL) was adopted to establish the standard curve according to chromogenic reaction based on 5% of sodium nitrite solution, 10% of aluminium nitrate solution and 4% of sodium hydroxide solution in 25 mL volumetric flasks, the obtained curve was used for the measurement of TF.

Swertiamarin curve was carried out with some modifications (Wan et al., 2021): Various volumes of swertiamarin solution (1.990 mg/mL) were added in 10 mL test tubes, then 0.4 mL of 5% vanillin in glacial acetic acid solution and 0.8 mL of perchloric acid were successively added and reacting for 20 min at 60 °C in a water bath. Next, the solution was cooling for 2 min in ice bath and diluted with acetic acid to 5 mL. Finally, the swertiamarin curve was gained and applied for the calculation of TP.

Gallic acid curve was conducted with some modifications (Martins, Monteiro, Do, & Da, 2021): Multiple volumes of gallic acid solution (0.1012 mg/mL) were added in 25 mL volumetric flasks, then 1 mL of folin phenol and 2 mL of 10% of natrium carbonicum solution were severally added and reacting for 60 min in dark. At last, the gallic acid curve was built and employed for the determination of TA.

#### 2.3.2 Single-factor investigation and response surface design

Three factors covered ethanol concentration (40%, 50%, 60%, 70%, 80%, 90%), liquid-to-solid ratio (10,1, 20:1, 30:1, 40:1, 50:1, 60:1), ultrasonic time (10 min, 20 min, 30 min, 40 min, 50 min, 60 min) were investigated respectively. Then, a three-factor three-level design model was carried out to further optimized the combination of ethanol concentration (A), liquid-to-solid ratio (B), and ultrasonic time (C). The yields of TF, TP, and TA were selected as the responses. The follow formula (1) relied on Design Expert 12.0 software was employed to conduct multiple regression analysis.

(1) $$Y=\beta_{0}+\sum_{i=1}^{k}\beta_{i}X_{i}+\sum_{i=1}^{k}\beta_{\mathrm{ii}}X_{i}^{2}+\sum_{i=1}^{k-1}\;\sum_{j=i+1}^{k}\beta_{\mathrm{ij}}X_{i}X_{j}$$

Where, Y represents the predicted yield. β_0_ is the constant coefficient. β_i_, β_ii_, and β_ij_ are the coefficients of linear, quadratic term, and interaction. X_i_ and X_j_ are the coded values of independent variables (i ≠ j). K is the number of independent variables.

#### 2.3.3 Confirmatory experiments

The optimal technologies of TF, TP, and TA were obtained based on the aforementioned experiments. In view of the convenience of subsequent operations, the screened parameters were modified and then three times of verification assays were carried out. Finally, the optimal technologies for industrial production were verified.

### 2.4 Location of candidate chemical indicators *via* serum pharmacochemistry analysis

#### 2.4.1 Sample preparation for administration

180 g of *Lonicera japonica* powder was extracted according to the optimal technology. After filtered, the solution was concentrated in vacuum to 1.0 g/mL. The obtained sample was used for animal experiment.

#### 2.4.2 Animal handing

After feeding for one week, Sprague-Dawley rats were randomly divided into blank group and administration group. Rats in administration group were given *Lonicera japonica* extract at a dose of 5 mL/kg for 9 days. After the last administration, all rats were anesthetized by pentobarbital sodium solution through intraperitoneal injection with a dose of 40 mg/kg body weight, blood was collected from abdominal aorta at different time points (15 min, 30 min, 60 min, 90 min, and 120 min), then the rats were sacrificed subsequently (euthanized *via* cervical dislocation under deep anesthesia). After placing for 2 h, the blood was centrifuged at 4000 r/min for 10 min, then the supernatant was separated.

#### 2.4.3 Serum processing

200 μL of serum from each group was taken out and 8 μL of phosphoric acid was added. After vortexing, the mixture was standing for 10 min, the operations were performed twice. Then, 4 times of methanol was added and mixed well. After centrifuged at 12000 r/min for 5 min at 4 °C, 800 μL of supernatant fitltered through a 0.22 μm microporous membrane was analyzed by UHPLC-HRMS. Besides, the foregoing administrated sample was as well as diluted for data collection synchronously.

#### 2.4.4 UHPLC-HRMS parameters under IDA mode

The mobile phase procedures were adopted as follows: 0–3 min, 5–15% A; 3–3.5 min, 15–15% A; 3.5–6 min, 15–30% A; 6–6.5 min, 30–30% A; 6.5–9 min, 30–50% A; 9–10 min, 50–50% A;10–15 min, 50–75% A; 15–20 min, 75–100% A. Other parameters were in accordance with the before-mentioned UHPLC-HRMS conditions under IDA mode.

#### 2.4.5 Location of candidate chemical indicators

Through peak processing, a matrix covered visual information was obtained. After non-target comparison of public database and the self-building database, compounds with high score and low mass error were screened out. Finally, ingredients appeared in administrated serum as well as in *Lonicera japonica* extract were deemed as the components absorbed into blood. Taking a comprehensive view of response values and structural features of the ingredients absorbed into blood, the bio-transformation processes *in vitro* and *in vivo* were supposed. Then the candidate chemical indicators of *Lonicera japonica* extract were screened out tentatively. The bio-activity of the candidate chemical indicators was further verified in cell.

### 2.5 Confirmation of final chemical indicators *via* anti-inflammatory evaluation

#### 2.5.1 Viability assay of RAW264.7 cells

RAW264.7 cells were cultured in an incubator at 37 °C with 5% CO_2_. Then cells in logarithmic growth period were seeded into 96-well plates with an appropriate concentration. After 24 h of incubation, the supernatant was discarded. 100 μL of DMEM contained candidate chemical indicators of different concentrations (1.56 μM, 3.13 μM, 6.25, μM 12.5 μM, 25 μM, 50 μM, 100 μM, 200 μM) were added to the corresponding wells simultaneously. After another 24 h of incubation, 10 μL of CCK-8 reagent was added to each well and reacting for 1 h in the incubator. Meantime, DMEM with cells was deemed as control group, DMEM was deemed as blank group. Then, the absorbance was detected at 450 nm using an enzyme detector. Finally, the cell viability rate of RAW264.7 cells were calculated by eq. (2).

(2) $$\text{Cell viability rate}\;\left(\%\right)=\left[\left(A_{\text{drug}}-A_{\text{blank}}\right)/\left(A_{\text{control}}-A_{\text{blank}}\right)\right]\times 100\%$$

where, A _drug_ was the absorbance of candidate chemical indicators; A _control_ was the absorbance of DMEM with cell; A _blank_ was the absorbance of DMEM.

#### 2.5.2 Measurement of NO production in RAW264.7 cells induced by LPS

Firstly, RAW264.7 cells were seeded into 96-well plates with an appropriate density and cultured for 24 h. After discarding supernatant, the wells were divided into different groups: 100 μL of DMEM was defined as control group; 100 μL of DMEM containing 1 μg/mL LPS was set as model control group, 100 μL of DMEM containing 50 μM dexamethasone and 1 μg/mL LPS was deemed as positive drug control group; different concentrations of candidate chemical indicators and 1 μg/mL LPS were named drug groups. After Every 24 h of incubation, 50 μL of supernatant was taken out and 50 μL of Griess reagent I and Griess reagent II were added orderly. Then, the absorbance was measured at 540 nm, and NO production was calculated using the corresponding standard curve. During the experiment, three parallels were conducted. At last, a one-way analysis of variance was carried out based on SPSS Statistics 26.0 software.

### 2.6 Application of chemical indicators in quality control of *Lonicera japonica* from different origins

#### 2.6.1 Sample preparation

Reference substance samples: Seventeen of standard substances covered chlorogenic acid (1), isochlorogenic acid A (2), isochlorogenic acid C (3), caffeic acid (4), neochlorogenic acid (5), luteolin-7-O-glucoside (6), hyperin (7), kaempfeol-3-O-rutinoside (8), luteolin (9), loniceroside (10), rutin(11), chrysoeriol (12), loganin (13), loganic acid (14), secoxyloganin (15), sweroside (16), and swertiamarin (17) were precisely weighed and dissolved in appropriate volume of methanol. Then the concentrations were maintained at 9.92 μg/mL, 59.52 μg/mL, 18.68 μg/mL, 4.08 μg/mL, 113.60 μg/mL, 7.98 μg/mL, 20.48 μg/mL, 17.48 μg/mL, 2.08 μg/mL, 16.84 μg/mL, 20.12 μg/mL, 1.460 μg/mL, 21.68 μg/mL, 20.48 μg/mL, 2.01 μg/mL, 20.16 μg/mL, and 80.96 μg/mL, respectively. After mixed with different proportions, series of mixing standard samples were obtained

*Lonicera japonica* samples: 0.5 g of *Lonicera japonica* from 18 of batches were weighed and extracting for 25 min under ultrasonic by 25 mL of 70% ethanol. After centrifuged at 12,000 r/min for 5 min, the supernatant was separated and passed through a 0.22 μm membrane for further analysis.

#### 2.6.2 UHPLC-HRMS conditions under MRM mode

The samples were analyzed through an UHPLC-HRMS under MRM mode. The gradient elution program based on T3 column (100 mm × 2.1 mm, 1.8 μm) was optimized as follows: 0–2.5 min, 5–20% A; 2.5–7.5 min, 20–20% A; 7.5–8 min, 20–100% A; 8–17 min, 100% A. The ions were detected by MRM under NEG at 5500 V. The individual standard sample was injected beforehand to screen the precursor ion, product ion, DP and CE.

#### 2.6.3 Linearity, precision, repeatability and stability assays

The linear curves were obtained according to concentrations and peaks' areas. In addition, the mixing standard solution was injected six times to gain the precision of instrument. *Lonicera japonica* sample was employed for analyzing the repeatability and stability (0 h, 6 h, 12 h, 18 h, 24 h, 48 h).

#### 2.6.4 Accumulated contents comparison and correlation analysis

Based on the established UHPLC-HRMS conditions under MRM mode, all the samples were analyzed. After handled with MultiQuant software, the contents of screened chemical indicators in *Lonicera japonica* from 18 batches were calculated. Finally, accumulated contents and correlation analysis were used for the general quality control of *Lonicera japonica*.

## 3 Results and discussion

### 3.1 Chemical identification of *Lonicera japonica*

**Fig. 1:**
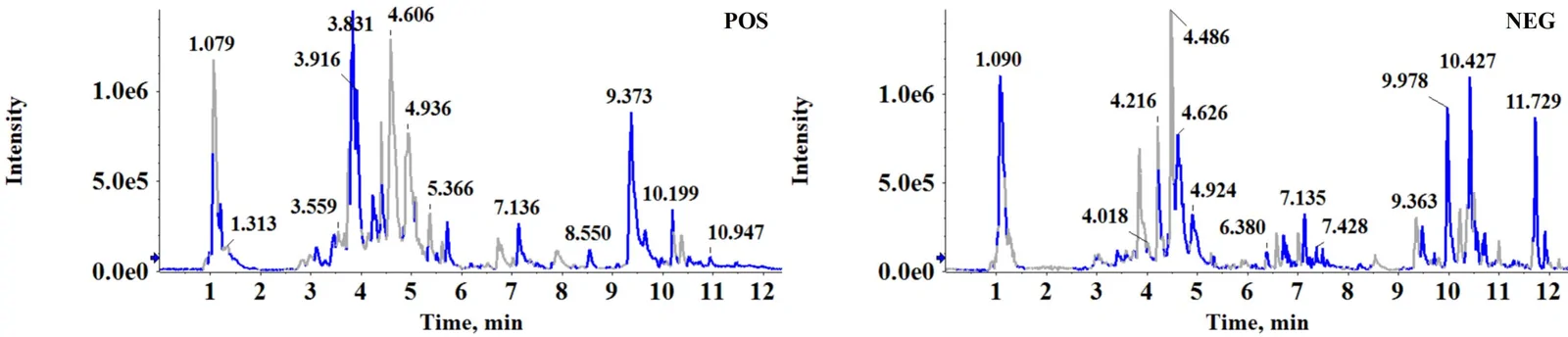
The base peak chromatograms of *Lonicera japonica* by UHPLC-HRMS under IDA mode for chemical understanding.

**Fig. 2:**
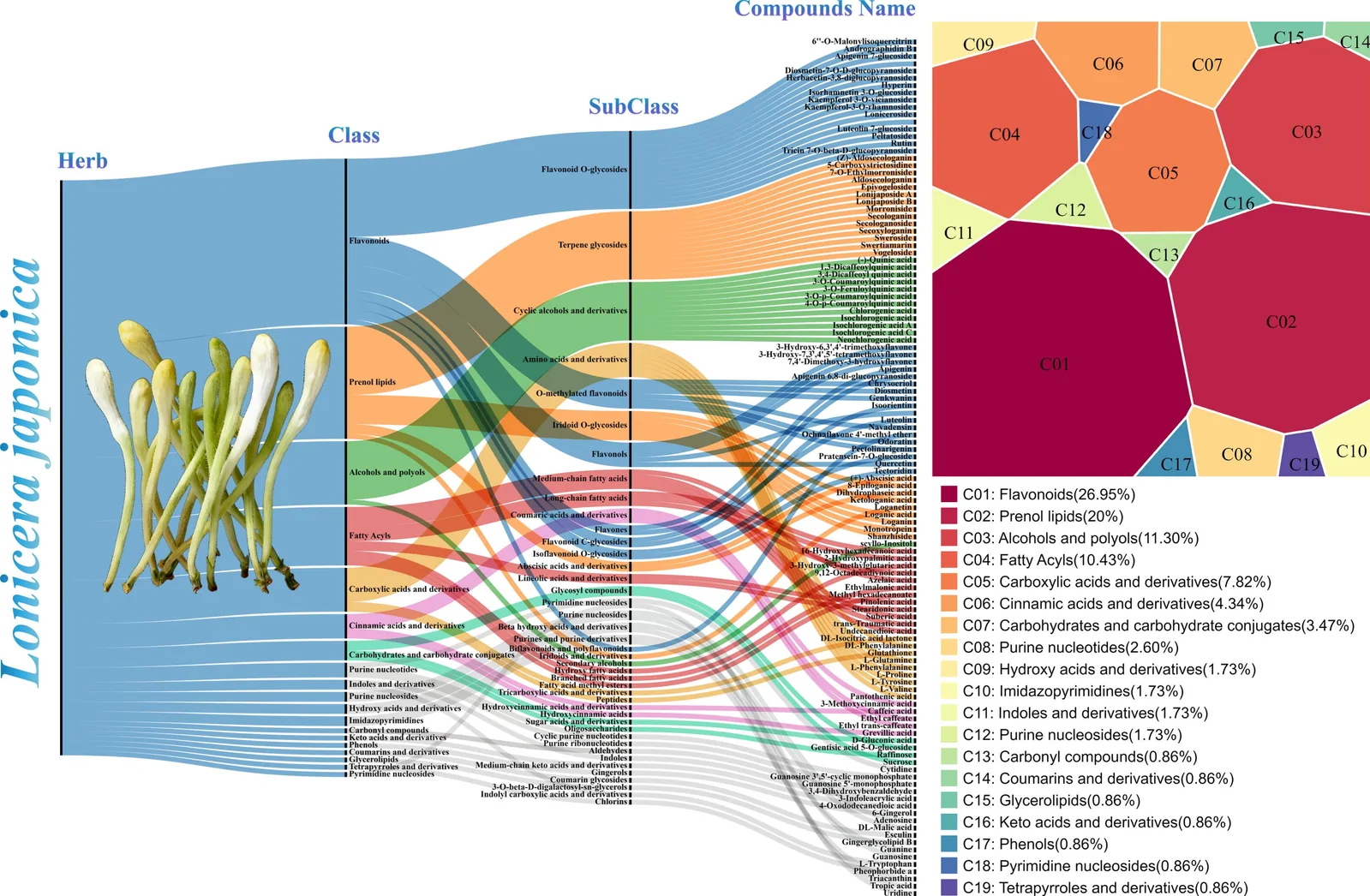
The classifications and proportions of identified ingredients in *Lonicera japonica*.

The base peak chromatograms (BPCs) of *Lonicera japonica* under POS and NEG were displayed in Fig. 1. The obtained BPCs showed that the established chromatographic conditions were applicative for the rapid chemical recognition. As for the screen of chemical indicators, global ingredients' explanation was especially important. Traditionally, sample for chemical analysis was extracted by specific solvent such as methanol (Chen et al., 2024), ethanol (Timotius, Rahayu, & Nurcahyanti, 2023) and water (Yang et al., 2023). However, the solubilities of various ingredients in one solvent are limited, resulted in the deficiency of unscrambling. Thus, sample covered full-scale information is vital for chemical understanding. Novel sample preprocessing based on gradient-solvent have been developed and tentatively applied in the global chemical understanding of some plants (Wang, Gu, Han, et al., 2025; Wang, Gu, Zhang, et al., 2025). After the characteristic sample preparation, totally 115 of components from *Lonicera japonica* were recognized. The detailed information of the identified ingredients were shown in Table 1. In addition, the chemical classifications and proportions were exhibited in Fig. 2. Flavonoids, prenol lipids, alcohols and polyols occupied top three, the proportions were 26.95%, 20.00%, and 11.30%. The result indicated that these three kinds were the important profiles of *Lonicera japonica*. Therefore, the extraction of TF, TP and TA were of particular importance for further study.

**Table 1:** The chemical identification results of *Lonicera japonica* by UHPLC-HRMS under IDA mode.

No.	Rt(min)	*M*/*z*(Da)	CAS	Name	Adduct type	Formula	Error(mDa)	Class
1	1.03	179.0578	488-59-5	scyllo-Inositol	[M − H] ^−^	C_6_H_12_O_6_	2.22	Alcohols and polyols
2	1.09	116.0705	147-85-3	L-Proline	[M + H] ^+^	C_5_H_9_NO_2_	− 0.69	Carboxylic acids and derivatives
3	1.09	147.0757	56-85-9	l-Glutamine	[M + H] ^+^	C_5_H_10_N_2_O_3_	− 1.30	Carboxylic acids and derivatives
4	1.09	191.0562	77-95-2	(−)-Quinic acid	[M − H] ^−^	C_7_H_12_O_6_	0.60	Alcohols and polyols
5	1.12	387.1147	57-50-1	Sucrose	[M + HCOO] ^−^	C_12_H_22_O_11_	0.80	Carbohydrates and carbohydrate conjugates
6	1.14	549.1668	512-69-6	Raffinose	[M + HCOO] ^−^	C_18_H_32_O_16_	0.13	Carbohydrates and carbohydrate conjugates
7	1.19	133.0153	6915-15-7	DL-Malic acid	[M − H] ^−^	C_4_H_6_O_5_	1.56	Hydroxy acids and derivatives
8	1.19	244.0928	65-46-3	Cytidine	[M + H] ^+^	C_9_H_13_N_3_O_5_	− 0.56	Purine nucleotides
9	1.21	173.0101	4702-32-3	DL-Isocitric acid lactone	[M − H] ^−^	C_6_H_6_O_6_	1.51	Carboxylic acids and derivatives
10	1.24	611.1437	27,025-41-8	Glutathione	[M − H] ^−^	C_20_H_32_N_6_O_12_S_2_	− 0.45	Carboxylic acids and derivatives
11	1.28	195.0507	526-95-4	D-Gluconic acid	[M − H] ^−^	C_6_H_12_O_7_	0.18	Carbohydrates and carbohydrate conjugates
12	1.38	204.1226	10,091-84-6	Triacanthin	[M + H] ^+^	C_10_H_13_N_5_	− 2.37	Imidazopyrimidines
13	1.50	118.0855	72-18-4	L-Valine	[M + H] ^+^	C_5_H_11_NO_2_	−1.20	Carboxylic acids and derivatives
14	1.73	487.1329	58-96-8	Uridine	[2 M − H] ^−^	C_9_H_12_N_2_O_6_	1.68	Pyrimidine nucleosides
15	1.78	180.0674	60-18-4	L-Tyrosine	[M − H] ^−^	C_9_H_11_NO_3_	1.34	Carboxylic acids and derivatives
16	1.87	344.0414	7665-99-8	Guanosine 3′,5′-cyclic monophosphate	[M − H] ^−^	C_10_H_12_N_5_O_7_P	1.83	Purine nucleotides
17	2.19	161.0470	503-49-1	3-Hydroxy-3-methylglutaric acid	[M − H] ^−^	C_6_H_10_O_5_	2.01	Fatty Acyls
18	2.39	268.1031 / 312.0971	58-61-7	Adenosine	[M + H] ^+^	C_10_H_13_N_5_O_4_	− 1.47 / 2.76	Purine nucleosides
19	2.49	364.0650	85-32-5	Guanosine 5′-monophosphate	[M + H] ^+^	C_10_H_14_N_5_O_8_P	− 0.86	Purine nucleotides
20	2.52	152.0564	73-40-5	Guanine	[M + H] ^+^	C_5_H_5_N_5_O	− 0.82	Imidazopyrimidines
21	2.52	284.0989 / 282.0833	118-00-3	Guanosine	[M + H] ^+^	C_10_H_13_N_5_O_5_	− 0.52 / − 0.51	Purine nucleosides
22	2.76	131.0356	601–75-2	Ethylmalonic acid	[M − H] ^−^	C_5_H_8_O_4_	1.13	Fatty Acyls
23	2.99	164.0725	63–91-2	L-Phenylalanine	[M − H] ^−^	C_9_H_11_NO_2_	1.35	Carboxylic acids and derivatives
24	3.03	391.1238	29,836–27-9	Shanzhiside	[M − H] ^−^	C_16_H_24_O_11_	− 0.26	Prenol lipids
25	3.05	166.0870	150–30-1	DL-Phenylalanine	[M + H] ^+^	C_9_H_11_NO_2_	0.22	Carboxylic acids and derivatives
26	3.15	435.1169	5945-50-6	Monotropein	[M + HCOO] ^−^	C_16_H_22_O_11_	3.04	Prenol lipids
27	3.18	315.0711	1820-89-9	Gentisic acid 5-O-glucoside	[M − H] ^−^	C_13_H_16_O_9_	− 0.53	Carbohydrates and carbohydrate conjugates
28	3.19	503.1863	1,016,988–36-5	Lonijaposide B	[M + H − 2H_2_O]^+^	C_25_H_32_NO_12_	7.12	Prenol lipids
29	3.22	218.1063	599–54-2	Pantothenic acid	[M − H] ^−^	C_9_H_17_NO_5_	3.43	Carboxylic acids and derivatives
30	3.31	373.1129	170,382–11-3	Ketologanic acid	[M − H] ^−^	C_16_H_22_O_10_	− 0.54	Prenol lipids
31	3.48	355.1023	906–33-2	Neochlorogenic acid	[M + H] ^+^	C_16_H_18_O_9_	− 0.61	Alcohols and polyols
32	3.57	188.0704	1204-06-4	3-Indoleacrylic acid	[M + H] ^+^	C_11_H_9_NO_2_	− 0.71	Indoles and derivatives
33	3.57	394.1718 / 375.1305	22,255–40-9	Loganic acid	[M + NH_4_] ^+^	C_16_H_24_O_10_	0.51 / 1.41	Prenol lipids
34	3.57	203.0852	73–22-3	L-Tryptophan	[M − H] ^−^	C_11_H_12_N_2_O_2_	3.15	Indoles and derivatives
35	3.58	179.0698	6099-04-3	3-Methoxycinnamic acid	[M + H] ^+^	C_10_H_10_O_3_	− 1.07	Cinnamic acids and derivatives
36	3.66	339.0750	531–75-9	Esculin	[M − H] ^−^	C_15_H_16_O_9_	3.35	Coumarins and derivatives
37	3.72	321.0961	1899-30-5	3-O-Coumaroylquinic acid	[M + H − H_2_O] ^+^	C_16_H_18_O_8_	− 1.38	Alcohols and polyols
38	3.75	625.1429	99,224–12-1	Herbacetin-3,8-diglucopyranoside	[M − H] ^−^	C_27_H_30_O_17_	2.39	Flavonoids
39	3.77	389.1100	59,472–23-0	Secologanoside	[M − H] ^−^	C_16_H_22_O_11_	1.59	Prenol lipids
40	3.78	424.1821 / 405.1381	25,406–64-8	Morroniside	[M + NH_4_] ^+^	C_17_H_26_O_11_	0.26 / − 1.5	Prenol lipids
41	3.86	355.1022 / 353.0863	327–97-9	Chlorogenic acid	[M + H] ^+^	C_16_H_18_O_9_	− 0.62 / −0.92	Alcohols and polyols
42	3.92	137.0249	139–85-5	3,4-Dihydroxybenzaldehyde	[M − H] ^−^	C_7_H_6_O_3_	1.05	Carbonyl compounds
43	3.93	149.0590	552–63-6	Tropic acid	[M + H − H_2_O] ^+^	C_9_H_10_O_3_	− 1.28	Hydroxy acids and derivatives
44	3.95	419.1193	17,388–39-5	Swertiamarin	[M + HCOO] ^−^	C_16_H_22_O_10_	0.35	Prenol lipids
45	4.10	593.1485	23,666–13-9	Apigenin 6,8-di-glucopyranoside	[M − H] ^−^	C_27_H_30_O_15_	− 2.11	Flavonoids
46	4.15	408.1868/435.1517	18,524–94-2	Loganin	[M + NH_4_] ^+^	C_17_H_26_O_10_	− 0.13 / 1.52	Prenol lipids
47	4.18	377.1456/421.1362	82,509–41-9	8-Epiloganic acid	[M + H] ^+^	C_16_H_24_O_10_	0.874 / 1.61	Prenol lipids
48	4.19	253.0719	102–37-4	Ethyl caffeate	[M + HCOO] ^−^	C_11_H_12_O_4_	0.64	Cinnamic acids and derivatives
49	4.20	181.0485	331–39-5	Caffeic acid	[M + H] ^+^	C_9_H_8_O_4_	− 1.60	Cinnamic acids and derivatives
50	4.21	179.0357	636–01-1	Grevillic acid	[M − H] ^−^	C_9_H_8_O_4_	1.23	Cinnamic acids and derivatives
51	4.25	335.0781	534–61-2	Isochlorogenic acid	[M − H_2_O − H] ^−^	C_16_H_18_O_9_	1.43	Alcohols and polyols
52	4.26	359.1329/357.1178	14,215–86-2	Sweroside	[M + H] ^+^	C_16_H_22_O_9_	− 1.23 / − 0.72	Prenol lipids
53	4.30	339.1071	53,539–37-0	4-O-p-Coumaroylquinic acid	[M + H] ^+^	C_16_H_18_O_8_	− 0.92	Alcohols and polyols
54	4.37	281.1372	41,756–77-8	Dihydrophaseic acid	[M − H] ^−^	C_15_H_22_O_5_	− 1.66	Prenol lipids
55	4.39	229.1071	29,748–10-5	Loganetin	[M + H] ^+^	C_11_H_16_O_5_	− 0.48	Prenol lipids
56	4.42	563.1971/561.1849	1,016,988–21-8	Lonijaposide A	[M + H − H_2_O] ^+^	C_27_H_34_NO_13_	− 3.11 / 0.31	Prenol lipids
57	4.43	449.1086	4261-42-1	Isoorientin	[M + H] ^+^	C_21_H_20_O_11_	0.19	Flavonoids
58	4.44	403.1254	58,822–47-2	Secoxyloganin	[M − H] ^−^	C_17_H_24_O_11_	1.34	Prenol lipids
59	4.48	369.1162/367.1045	62,929–69-5	3-O-Feruloylquinic acid	[M + H] ^+^	C_17_H_20_O_9_	− 2.31 / 1.66	Alcohols and polyols
60	4.53	595.1308	23,284–18-6	Peltatoside	[M − H] ^−^	C_26_H_28_O_16_	0.85	Flavonoids
61	4.57	337.0945	5746-55-4	3-O-p-Coumaroylquinic acid	[M − H] ^−^	C_16_H_18_O_8_	2.14	Alcohols and polyols
62	4.61	579.1352	110,352–79-9	Kaempferol 3-O-vicianoside	[M − H] ^−^	C_26_H_28_O_15_	0.20	Flavonoids
63	4.62	389.1444	19,351–63-4	Secologanin	[M + H] ^+^	C_17_H_24_O_10_	− 0.36	Prenol lipids
64	4.63	406.1687	118,627–52-4	Epivogeloside	[M + NH_4_] ^+^	C_17_H_24_O_10_	− 2.64	Prenol lipids
65	4.64	611.1593/609.1515	153–18-4	Rutin	[M + H] ^+^	C_27_H_30_O_16_	− 1.82 / 5.93	Flavonoids
66	4.71	593.1494	25,694–72-8	Loniceroside	[M − H] ^−^	C_27_H_30_O_15_	− 1.28	Flavonoids
67	4.71	595.1676	17,650–84-9	Kaempfeol-3-O-rutinoside	[M + H] ^+^	C_27_H_30_O_15_	1.27	Flavonoids
68	4.75	575.2216	34,371–11-4	5-Carboxystrictosidine	[M + H] ^+^	C_28_H_34_N_2_O_11_	− 2.46	Prenol lipids
69	4.75	465.1039/463.0867	482–36-0	Hyperin	[M + H] ^+^	C_21_H_20_O_12_	0.634 / − 0.88	Flavonoids
70	4.77	173.0840	505–48-6	Suberic acid	[M − H] ^−^	C_8_H_14_O_4_	2.63	Fatty Acyls
71	4.77	449.1062/447.0923	5373-11-5	Luteolin-7-O-glucoside	[M + H] ^+^	C_21_H_20_O_11_	− 2.16 / − 0.35	Flavonoids
72	4.83	549.0894	96,862–01-0	6”-O-Malonylisoquercitrin	[M − H] ^−^	C_24_H_22_O_15_	1.34	Flavonoids
73	4.87	757.2569	471,271–55-3	Aldosecologanin	[M − H] ^−^	C_34_H_46_O_19_	1.35	Prenol lipids
74	4.88	776.2954	82,474–97-3	(*Z*)-Aldosecologanin	[M + NH_4_] ^+^	C_34_H_46_O_19_	− 2.35	Prenol lipids
75	4.93	499.1261	19,870–46-3	1,3-Dicaffeoylquinic acid	[M + H − H_2_O] ^+^	C_25_H_24_O_12_	2.04	Alcohols and polyols
76	4.94	1031.2524	32,451–88-0	Isochlorogenic acid C	[2 M − H] ^−^	C_25_H_24_O_12_	6.64	Alcohols and polyols
77	4.94	479.1201	5041-82-7	Isorhamnetin 3-O-glucoside	[M + H] ^+^	C_22_H_22_O_12_	1.14	Flavonoids
78	4.94	517.1358/515.1204	2450-53-5	Isochlorogenic acid A	[M + H] ^+^	C_25_H_24_O_12_	1.24 / 1.45	Alcohols and polyols
79	4.99	431.0980	578–74-5	Apigenin 7-glucoside	[M − H] ^−^	C_21_H_20_O_10_	0.20	Flavonoids
80	5.00	463.1245	20,126–59-4	Diosmetin-7-O-D-glucopyranoside	[M + H] ^+^	C_22_H_22_O_11_	0.44	Flavonoids
81	5.03	461.1101	611–40-5	Tectoridin	[M − H] ^−^	C_22_H_22_O_11_	1.73	Flavonoids
82	5.06	369.1207	60,077–47-6	Vogeloside	[M − H_2_O − H] ^−^	C_17_H_24_O_10_	2.16	Prenol lipids
83	5.12	187.0979	123–99-9	Azelaic acid	[M − H] ^−^	C_9_H_16_O_4_	0.87	Fatty Acyls
84	5.14	531.1484	114,637–83-1	3,4-Dicaffeoyl quinic acid	[M + H] ^+^	C_26_H_26_O_12_	− 1.89	Alcohols and polyols
85	5.22	243.1236	30,828–09-2	4-Oxododecanedioic acid	[M − H] ^−^	C_12_H_20_O_5_	0.37	Keto acids and derivatives
86	5.29	417.1774	945,721–10-8	7-O-Ethylmorroniside	[M + H − H_2_O] ^+^	C_19_H_30_O_11_	1.32	Prenol lipids
87	5.35	287.0553/285.0426	491–70-3	Luteolin	[M + H] ^+^	C_15_H_10_O_6_	− 0.17 / 2.76	Flavonoids
88	5.35	461.1129	36,191–03-4	Pratensein-7-O-glucoside	[M − H] ^−^	C_22_H_22_O_11_	4.54	Flavonoids
89	5.38	263.1282	21,293–29-8	(+)-Abscisic acid	[M − H] ^−^	C_15_H_20_O_4_	− 0.14	Prenol lipids
90	5.38	301.0351	117–39-5	Quercetin	[M − H] ^−^	C_15_H_10_O_7_	0.26	Flavonoids
91	5.39	493.1344	32,769–01-0	Tricin 7-O-beta-D-glucopyranoside	[M + H] ^+^	C_23_H_24_O_12_	− 0.23	Flavonoids
92	5.42	491.1224	113,963–38-5	Andrographidin B	[M − H] ^−^	C_23_H_24_O_12_	3.47	Flavonoids
93	5.51	207.0666	66,648–50-8	Ethyl trans-caffeate	[M − H] ^−^	C_11_H_12_O_4_	0.84	Cinnamic acids and derivatives
94	5.55	271.0596/269.0464	520–36-5	Apigenin	[M + H] ^+^	C_15_H_10_O_5_	− 0.96 / 1.45	Flavonoids
95	5.55	215.1282	1852-04-6	Undecanedioic acid	[M − H] ^−^	C_11_H_20_O_4_	− 0.11	Fatty Acyls
96	5.61	301.0698	491–71-4	Chrysoeriol	[M + H] ^+^	C_16_H_12_O_6_	− 1.42	Flavonoids
97	5.62	299.0559	520–34-3	Diosmetin	[M − H] ^−^	C_16_H_12_O_6_	0.36	Flavonoids
98	5.65	227.1269	6402-36-4	trans-Traumatic acid	[M − H] ^−^	C_12_H_20_O_4_	− 1.45	Fatty Acyls
99	6.11	283.0598	437–64-9	Genkwanin	[M − H] ^−^	C_16_H_12_O_5_	− 0.81	Flavonoids
100	6.15	343.0813	10,176–66-6	Navadensin	[M − H] ^−^	C_18_H_16_O_7_	− 0.44	Flavonoids
101	6.17	293.1770	23,513–14-6	6-Gingerol	[M − H] ^−^	C_17_H_26_O_4_	1.72	Phenols
102	6.18	315.0864	520–12-7	Pectolinarigenin	[M + H] ^+^	C_17_H_14_O_6_	− 0.49	Flavonoids
103	6.19	313.0738	53,948–00-8	Odoratin	[M − H] ^−^	C_17_H_14_O_6_	2.61	Flavonoids
104	6.40	551.0950	49,619–87-6	Ochnaflavone 4′-methyl ether	[M − H] ^−^	C_31_H_20_O_10_	− 2.82	Flavonoids
105	6.59	329.1021	3518-24-9	3-Hydroxy-6,3′,4′-trimethoxyflavone	[M + H] ^+^	C_18_H_16_O_6_	− 0.45	Flavonoids
106	6.66	723.3805	88,168–90-5	Gingerglycolipid B	[M + HCOO] ^−^	C_33_H_58_O_14_	0.18	Glycerolipids
107	6.75	275.2011	20,290–75-9	Stearidonic acid	[M − H] ^−^	C_18_H_28_O_2_	0.00	Fatty Acyls
108	6.75	277.2164	2012-14-8	9,12-Octadecadiynoic acid	[M + H] ^+^	C_18_H_28_O_2_	− 0.36	Fatty Acyls
109	6.76	359.1120	830,348–41-9	3-Hydroxy-7,3′,4′,5′-tetramethoxyflavone	[M + H] ^+^	C_19_H_18_O_7_	− 1.04	Flavonoids
110	6.77	315.2549	112–39-0	Methyl hexadecanoate	[M + HCOO] ^−^	C_17_H_34_O_2_	1.33	Fatty Acyls
111	6.93	299.0909	13,198–99-7	7,4’-Dimethoxy-3-hydroxyflavone	[M + H] ^+^	C_17_H_14_O_5_	− 1.02	Flavonoids
112	6.97	271.2269	506–13-8	16-Hydroxyhexadecanoic acid	[M − H] ^−^	C_16_H_32_O_3_	− 0.42	Fatty Acyls
113	7.14	279.2328	16,833–54-8	Pinolenic acid	[M + H] ^+^	C_18_H_30_O_2_	0.38	Fatty Acyls
114	9.43	271.2279	764–67-0	2-Hydroxypalmitic acid	[M − H] ^−^	C_16_H_32_O_3_	0.58	Fatty Acyls
115	10.44	593.2756/591.2641	15,664–29-6	Pheophorbide a	[M + H] ^+^	C_35_H_36_N_4_O_5_	− 0.75/3.40	Tetrapyrroles and derivatives

### 3.2 Optimum technologies of TF, TP and TA

#### 3.2.1 Standard curves of rutin, gallic acid and swertiamarin

**Fig. 3:**
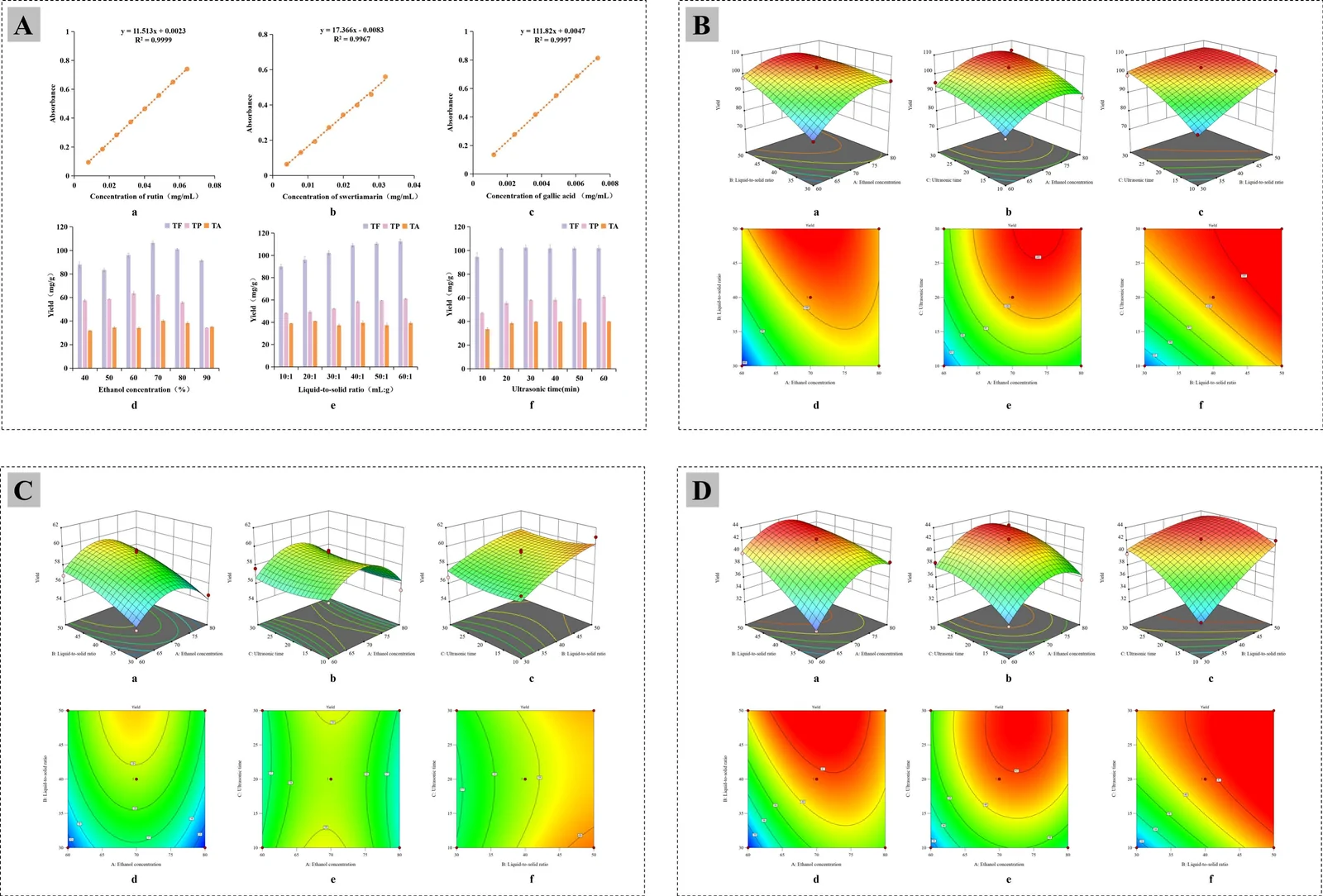
The standard curves, single-factor effects, surface plots and contour maps of TF, TP and TA from *Lonicera japonica*. A-(a–c): The standard curve of rutin, swertiamarin and gallic acid; A-(d–f): The effects of ethanol concentration, liquid-to-solid and ultrasonic time on the yields of TF, TP and TA. B-(a–f): The surface plots and contour maps of TF; C-(a–f): The surface plots and contour maps of TP; D-(a–f): The surface plots and contour maps of TA.

UV–visible spectrum was a common technology to detect solution shows characteristic UV/visible absorption spectra (Huang et al., 2025). Linear regressions of rutin, gallic acid and swertiamarin were plotted with concentration (X) and absorbance (Y), the standard curves were shown in Fig. 3-A-a, Fig. 3-A-b and Fig. 3-A-c. The approving linear scopes of rutin, swertiamarin and gallic acid were 0.01–0.07 mg/mL, 0.003–0.03 mg/mL, and 0.001–0.008 mg/mL. The established standard curves were employed for the calculation of TF, TP and TA in *Lonicera japonica*.

#### 3.2.2 Influences of single-factor

Three factors covered ethanol concentration, liquid-to-solid ratio and ultrasonic time were screened in sequence and the results were shown in Fig. 3-A-d, Fig. 3-A-e and Fig. 3-A-f. The yields of TF, TP and TA were increased gradually and then reached to blance at 70% ethanol. Therefore, 70% ethanol was selected for extracting TF, TP and TA. Liquid-to-solid ratio played vital role in solution viscosity, molecular interaction, and cavitation threshold (Yang, Li, Wang, Chang, & Jiang, 2021), As for TF and TP, the yields were fleetly rise until the liquid-to-solid ratio at 40:1, indicating that 40:1 was the favourable liquid-to-solid ratio to extract TF and TP. However, as for TA, the yield during 10:1 to 60:1 showed no significant change, manifesting that liquid-to-solid ratio during 10:1 to 60:1 could extract TA satisfactorily. Therefore, in order to extract TF, TP and TA simultaneously, the pleasing liquid-to-solid ratio was chose at 40:1. When referred to the influence of ultrasonic time, the yields of TF, TP and TA increased significantly within 10 min to 20 min, and then showed steady trend after 20 min. Hence, the ultrasonic time to extract TF, TP and TA concurrently was selected at 20 min.

#### 3.2.3 Response surface experiment results

Based on the single-factor experiments, three response surfaces of TF, TP and TA were designed and the results were displayed in Table 2. Besides, multiple regression analysis was conducted and the results of variance analysis were shown in Table 3. The regression equations of TF, TP and TA were as follows: Y _(TF)_ = 101.43 + 4.72A + 5.92B + 6.28C − 4.01AB + 0.1743AC − 3.43BCE − 6.65A^2^–1.26B^2^–2.76C^2^, Y _(TP)_ = 58.73–0.1592A + 1.41B − 0.0275C − 0.0076AB + 0.2783AC − 0.19BCE − 2.42A^2^–0.5106B^2^–0.4477C^2^, Y _(TA)_ = 41.57 + 1.77A + 2.81B + 2.28C − 1.62AB + 0.0774AC − 1.41BCE − 3.15A^2^–0.7752B^2^–1.29C^2^.

**Table 2:** Response surface experimental design and the yields of TF, TP and TA from *Lonicera japonica*.

No.	Ethanol concentration (%)	Solid-liquid ratio (g:mL)	Ultrasonic time (min)	TF (mg/g)	TP (mg/g)	TA (mg/g)
1	60	30	20	78.82	54.22	31.09
2	80	30	20	97.21	54.73	38.53
3	60	50	20	97.86	56.88	39.98
4	80	50	20	100.20	57.36	40.96
5	60	40	10	80.05	56.98	32.93
6	80	40	10	88.22	55.29	35.64
7	60	40	30	95.48	57.67	38.46
8	80	40	30	104.35	57.09	41.48
9	70	30	10	82.23	57.66	33.58
10	70	50	10	102.52	61.03	41.98
11	70	30	30	99.17	56.69	39.85
12	70	50	30	104.98	59.30	42.61
13	70	40	20	103.86	57.72	42.21
14	70	40	20	100.72	59.62	41.21
15	70	40	20	100.17	58.21	40.91
16	70	40	20	101.05	58.66	42.25
17	70	40	20	101.36	59.44	41.26

**Table 3:** The result of variance analysis in the optimization of TF, TP and TA from *Lonicera japonica*.

Source	Sum of squares			Degree of freedom	Mean square			F-value			*P*-value		
	TF	TP	TA		TF	TP	TA	TF	TP	TA	TF	TP	TA
Model	1126.12	43.32	203.35	9	125.12	4.81	22.59	27.32	4.57	31.92	0.0001 ^⁎⁎^	0.0287 ^⁎^	< 0.0001 ^⁎⁎^
A-A	178.35	0.2029	24.98	1	178.35	0.2029	24.98	38.93	0.1928	35.3	0.0004 ^⁎⁎^	0.6738	0.0006 ^⁎⁎^
B-B	280.7	15.84	63.03	1	280.70	15.84	63.03	61.28	15.06	89.05	0.0001 ^⁎⁎^	0.0061 ^⁎⁎^	< 0.0001 ^⁎⁎^
C-C	315.13	0.006	41.75	1	315.13	0.006	41.75	68.8	0.0057	58.99	< 0.0001 ^⁎⁎^	0.9417	0.0001 ^⁎⁎^
AB	64.36	0.0002	10.44	1	64.36	0.0002	10.44	14.05	0.0002	14.75	0.0072 ^⁎⁎^	0.9885	0.0064 ^⁎⁎^
AC	0.1215	0.3097	0.024	1	0.12	0.31	0.02	0.03	0.29	0.03	0.8752	0.6043	0.8593
BC	47.14	0.1445	7.94	1	47.14	0.14	7.94	10.29	0.14	11.22	0.0149 ^⁎^	0.7219	0.0123 ^⁎^
A^2^	186.08	24.75	41.84	1	186.08	24.75	41.84	40.62	23.52	59.11	0.0004 ^⁎⁎^	0.0019 ^⁎⁎^	0.0001 ^⁎⁎^
B^2^	6.68	1.10	2.53	1	6.68	1.10	2.53	1.46	1.04	3.57	0.2665	0.3411	0.1006
C^2^	32.03	0.84	6.98	1	32.03	0.84	6.98	6.99	0.802	9.86	0.0332 ^⁎^	0.4002	0.0164 ^⁎^
Residual error	32.06	7.37	4.95	7	4.58	1.05	0.71						
Lack of Fit	23.9	4.77	3.41	3	7.97	1.59	1.14	3.9	2.94	2.45	0.1108	0.1623	0.2032
Pure error	8.17	2.59	1.55	4	2.04	0.65	0.39						
Total deviation	1158.18	50.68	208.31	16									
R^2^	TF: 0.9723; TP: 0.8547; TA: 0.9762												
Equations	Y _(TF)_ = 101.43 + 4.72A + 5.92B + 6.28C − 4.01AB + 0.1743AC − 3.43BCE − 6.65A^2^–1.26B^2^–2.76C^2^; Y _(TP)_ = 58.73–0.1592A + 1.41B − 0.0275C − 0.0076AB + 0.2783AC − 0.19BCE − 2.42A^2^–0.5106B^2^–0.4477C^2^; Y _(TA)_ = 41.57 + 1.77A + 2.81B + 2.28C − 1.62AB + 0.0774AC − 1.41BCE − 3.15A^2^–0.7752B^2^–1.29C^2^.												

* p < 0.05, significant, ** p < 0.01, extremely significant.

P, R^2^ and F values could show the detailed fitting status of established models (Kotik, Kulik, & Valentova, 2023). *P* values in models of TF, TP and TA were less than 0.05, indicating that the regression equation could exhibit the relationship between yield and experimental factor. In addition, P values of lack-of-fit in models of TF, TP and TA were greater than 0.05, indicating that the models showed well fitting degree in predicting the optimal extraction conditions. R^2^ in models of TF, TP and TA were 0.9723, 0.8547 and 0.9762, which showed that the experimental data and predicted data owned little differences. Through the comparison of F values, the factors' influencing orders were as follows. TF: C > B > A, TP: C > A > B, TA: B > C > A.

The response surface plots and interaction effects between two factors in the extraction of TF, TP, and TA were severally displayed in Fig. 3-B (a–f), Fig. 3-C (a–f) and Fig. 3-D (a–f). Generally speaking, the steepness of response surface and the oval degree of contour center are correlated with the interaction effect positively. As for TF model and TA model, the interaction effect of AB and BC in models of TF and TA were significant. As for TP model, the interaction effect of AB, AC and BC were not significant. These results were as well as verified in the variance analysis. The predicted yields of TF, TP and TA were 106.80 mg/g, 59.25 mg/g, and 43.75 mg/g under the optimal ethanol concentrations (TF:70.6%, TP: 75%, TA: 70.3%), liquid-to-solid ratio (TF: 50:1, TP: 50:1, TA: 50:1), and ultrasonic time (TF: 25.2 min, TP: 20 min, TA: 23.4 min). In view of the feasibility of experiment, the optimal extraction technologies of TF, TP and TA were uniformly modified as ethanol concentration of 70%, liquid-to-solid ratio of 50:1 and ultrasonic time of 25 min. Under the modified conditions, the yields of TF, TP and TA were 110.00 mg/g, 58.44 mg/g and 41.47 mg/g. The results were shown in Table 4. The result explained that the sufficient target ingredients in *Lonicera japonica* could be extracted based on the modified technology.

**Table 4:** The predicted and modified of extraction technologies of TF, TP and TA from *Lonicera japonica*.

Class	Prediction				Modification			
	Ethanol concentrations (%)	Solid-liquid ratio (g:mL)	Ultrasonic time (min)	Yield (mg/g)	Ethanol concentrations (%)	Solid-liquid ratio (g:mL)	Ultrasonic time (min)	Yield (mg/g)
TF	70.6	1:50	25.2	106.80	70	1:50	25	110.00
TP	75	1:50	20	59.25				58.44
TA	70.3	1:50	23.4	43.75				41.47

### 3.3 Targeted selection of candidate chemical indicators of *Lonicera japonica*

#### 3.3.1 Screening of constituents absorbed into blood

**Fig. 4:**
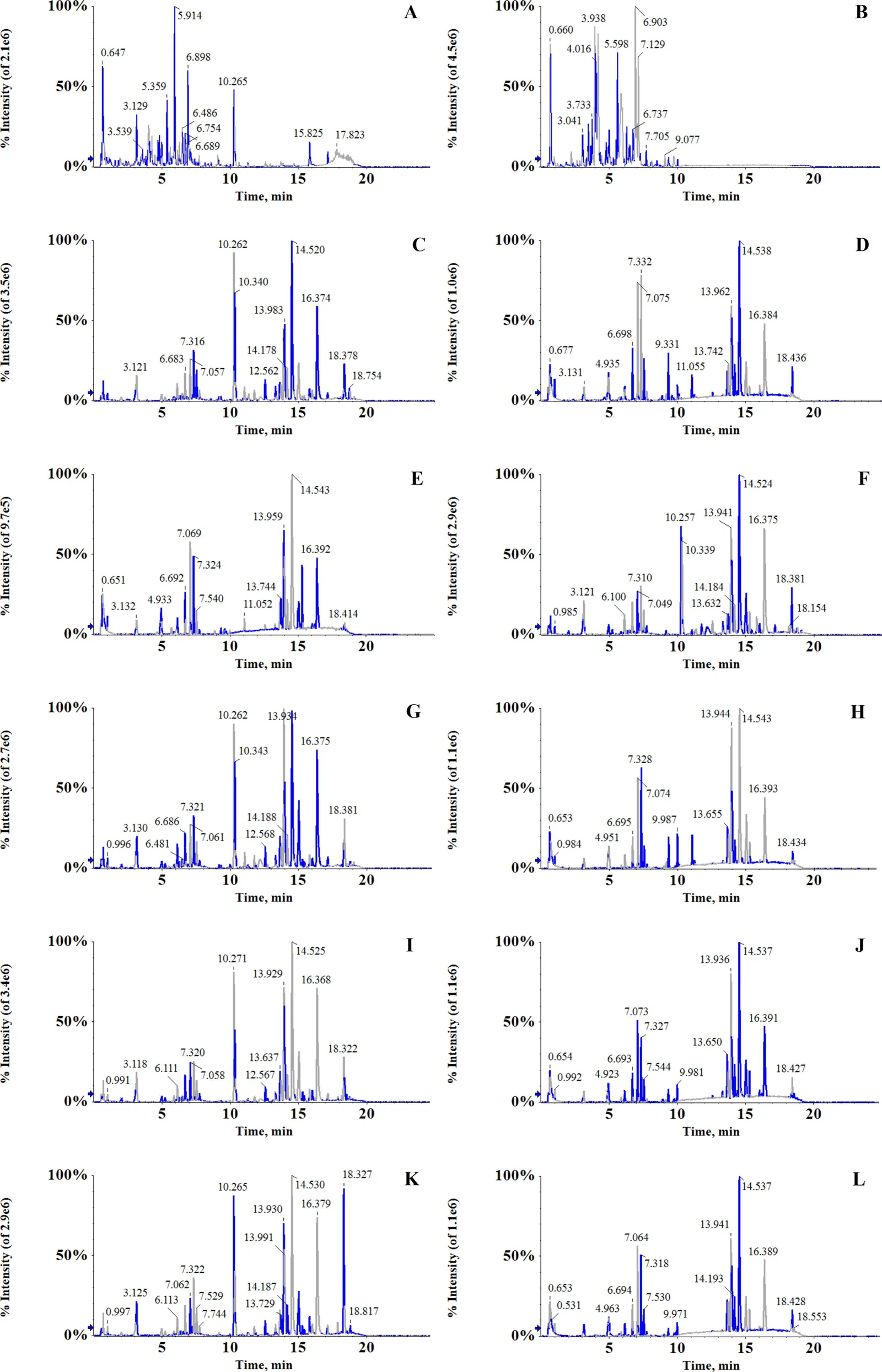
The base peak chromatograms of *Lonicera japonica* extract and dosed serum by UHPLC-HRMS under IDA mode for constituents absorbed into blood. A: The base peak chromatograms of *Lonicera japonica* extract under POS; B: The base peak chromatograms of *Lonicera japonica* extract under NEG; C: The base peak chromatograms of dosed serum at 15 min under POS; D: The base peak chromatograms of dosed serum at 15 min under NEG; E: The base peak chromatograms of dosed serum at 30 min under POS; F: The base peak chromatograms of dosed serum at 30 min under NEG; G: The base peak chromatograms of dosed serum at 60 min under POS; H: The base peak chromatograms of dosed serum at 60 min under NEG; I: The base peak chromatograms of dosed serum at 90 min under POS; J: The base peak chromatograms of dosed serum at 90 min under NEG; K: The base peak chromatograms of dosed serum at 120 min under POS; L: The base peak chromatograms of dosed serum at 120 min under NEG.

In order to obtain well-pleasing separation for the screen of constituents absorbed into blood, the chromatographic conditions were ulteriorly optimized. The BPCs of *Lonicera japonica* extract and serum sample were shown in Fig. 4. The detected ions were abundant and separated well, meaning that the established conditions were applicable for further analysis.

**Fig. 5:**
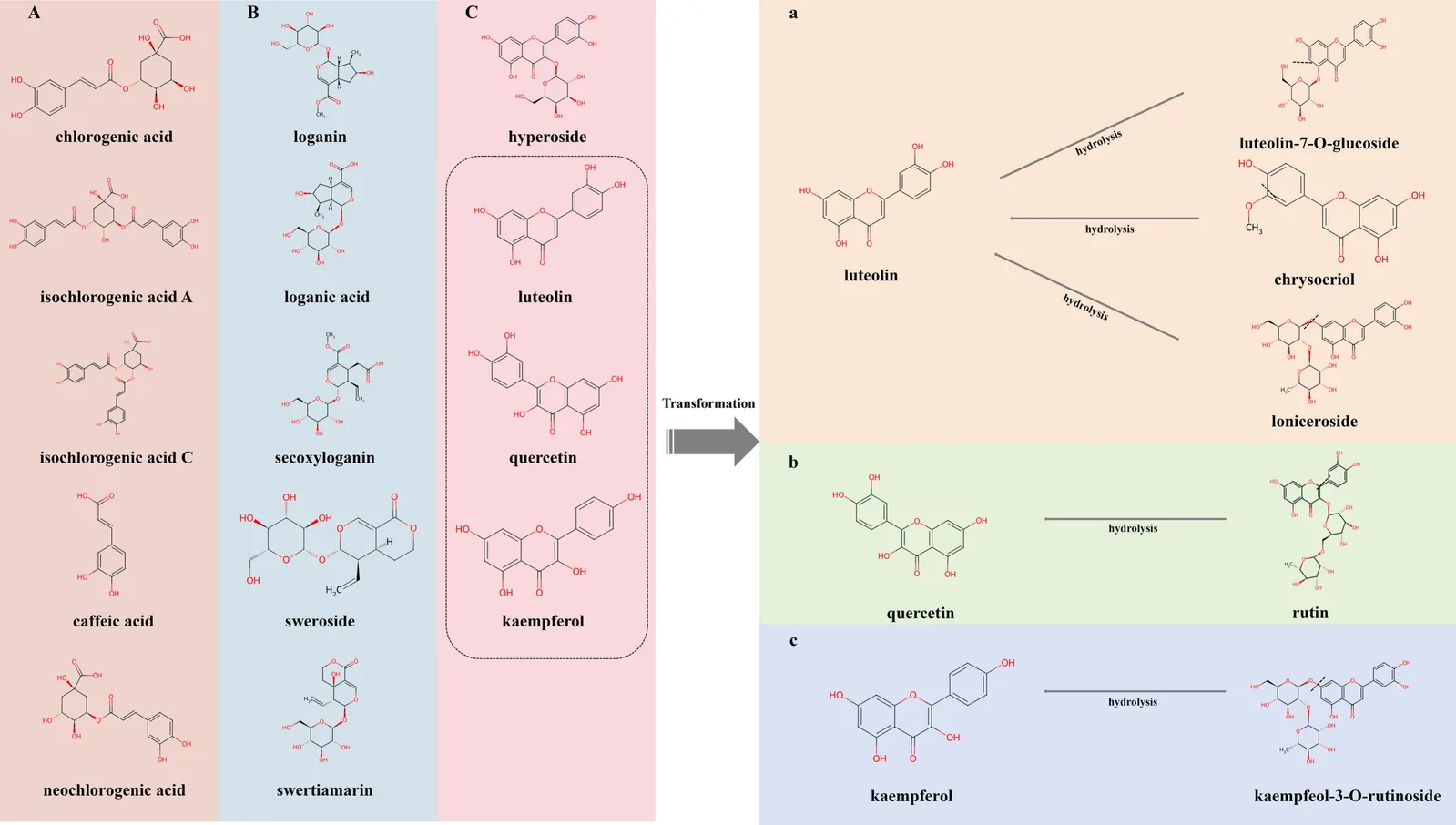
The constituents absorbed into blood and the possible transformation processes among flavonoid glycosides and their aglycones. A: The alcohols and polyols absorbed into blood; B: The prenol lipids absorbed into blood; C: The flavonoids absorbed into blood. a: The transformation processes among luteolin-7-O-glucoside, chrysoeriol, loniceroside and luteolin; b: The transformation process between rutin and quercetin; c: The transformation process between kaempfeol-3-O-rutinoside and kaempferol.

Through the ingredients' analysis *in vivo*, 14 of ingredients with strong responses were synchronously located from the dosed serum, covered 5 of alcohols and polyols (chlorogenic acid, isochlorogenic acid A, isochlorogenic acid C, caffeic acid, neochlorogenic acid), 5 of prenol lipids (loganin, loganic acid, secoxyloganin, sweroside, swertiamarin) and 4 of flavonoids (luteolin, hyperin, quercetin, kaempferol). The results were displayed in Fig. 5-A, -B and -C.

Nevertheless, the process of chemical components *in vivo* is complex, metabolism and transport might be occurred (Wu et al., 2024). As for flavonoids, the transformation between glycoside and aglycone *in vitro* and *in vivo* have been reported (Kotik et al., 2023). Hence, the screened flavonoid indicators should be reconfirmed based on response intensities. Luteolin showed high intensity not only *in vitro* but also *in vivo*, explained that luteolin could be deemed as a candidate indicator. Except that, three flavonoid glycosides (luteolin-7-O-glucoside, chrysoeriol and loniceroside), based on luteolin aglycone showed high response intensities *in vitro,* they might losing glycosyls and then transformed into luteolin *in vivo* (Li et al., 2017)*.* Therefore, luteolin-7-O-glucoside, chrysoeriol and loniceroside could as well as added as the candidate indicators. As for the absorbed constituents named quercetin and kaempferol, the response intensities *in vitro* were particularly low. This indicated that the detected ions *in vivo* may derived from their flavonoid glycosides named rutin and kaempfeol-3-O-rutinoside severally (Aldian et al., 2025; Feng, Li, Brobbey Oppong, & Qiu, 2018), which exhibited high response intensities *in vitro.* Hence, in view of the transforming relationships as well as the response intensities, rutin and kaempfeol-3-O-rutinoside are more reasonable to selected as the candidate indicators instead of quercetin and kaempferol. The possible transformation processes were displayed in Fig. 5-a, -b and -c.

Summing up the above, totally 17 of chemical profiles were selected as the candidate chemical indicators of *Lonicera japonica* preliminarily, including chlorogenic acid (1), isochlorogenic acid A (2), isochlorogenic acid C (3), caffeic acid (4), neochlorogenic acid (5), luteolin-7-O-glucoside (6), hyperin (7), kaempfeol-3-O-rutinoside (8), luteolin (9), loniceroside (10), rutin (11), chrysoeriol (12), loganin (13), loganic acid (14), secoxyloganin (15), sweroside (16), and swertiamarin (17). The biological activity of these nominated indicators need furtherly evaluated in cell experiment.

#### 3.3.2 Cell viability and anti-inflammatory activity

**Fig. 6:**
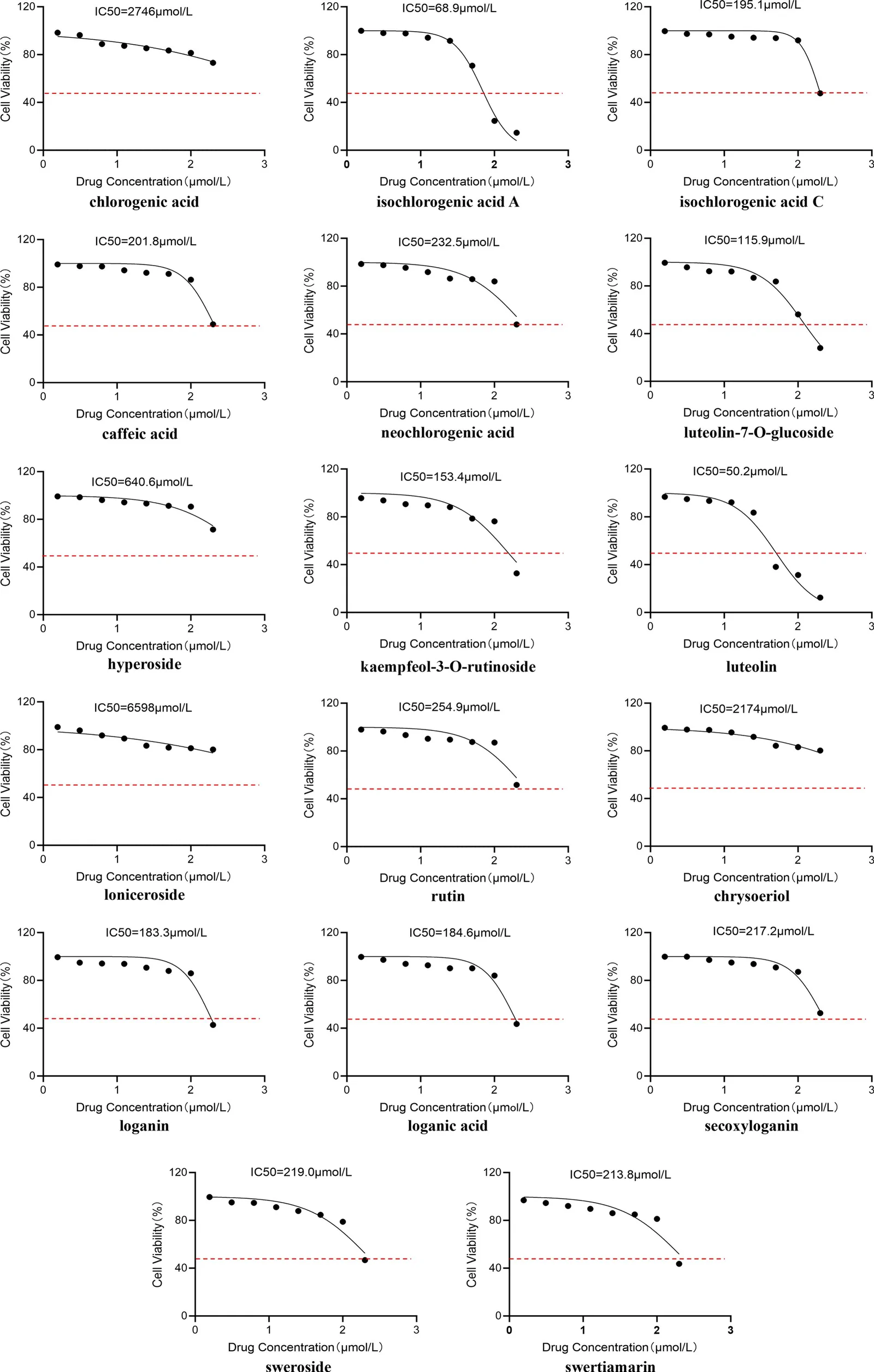
Cell viability of RAW264.7 influenced by candidate chemical indicators.

The results of cell viability influenced by 17 candidate chemical indicators were displayed in Fig. 6. All of the candidate chemical indicators showed wide safety ranges. Anti-inflammatory is one characteristic biological activity of *Lonicera japonica* (Li, Li, Fu, Song, & Fu, 2020). In consequence, anti-inflammatory evaluation of nominated chemical indicators was helpful for the final confirmation of chemical indicators. Generally, NO production of RAW264.7 cells was a classic index to evaluate the effect of anti-inflammatory (Hou et al., 2024; Tan et al., 2025). Hence, three concentrations of candidate chemical indicators were selected to measure the NO production.

**Fig. 7:**
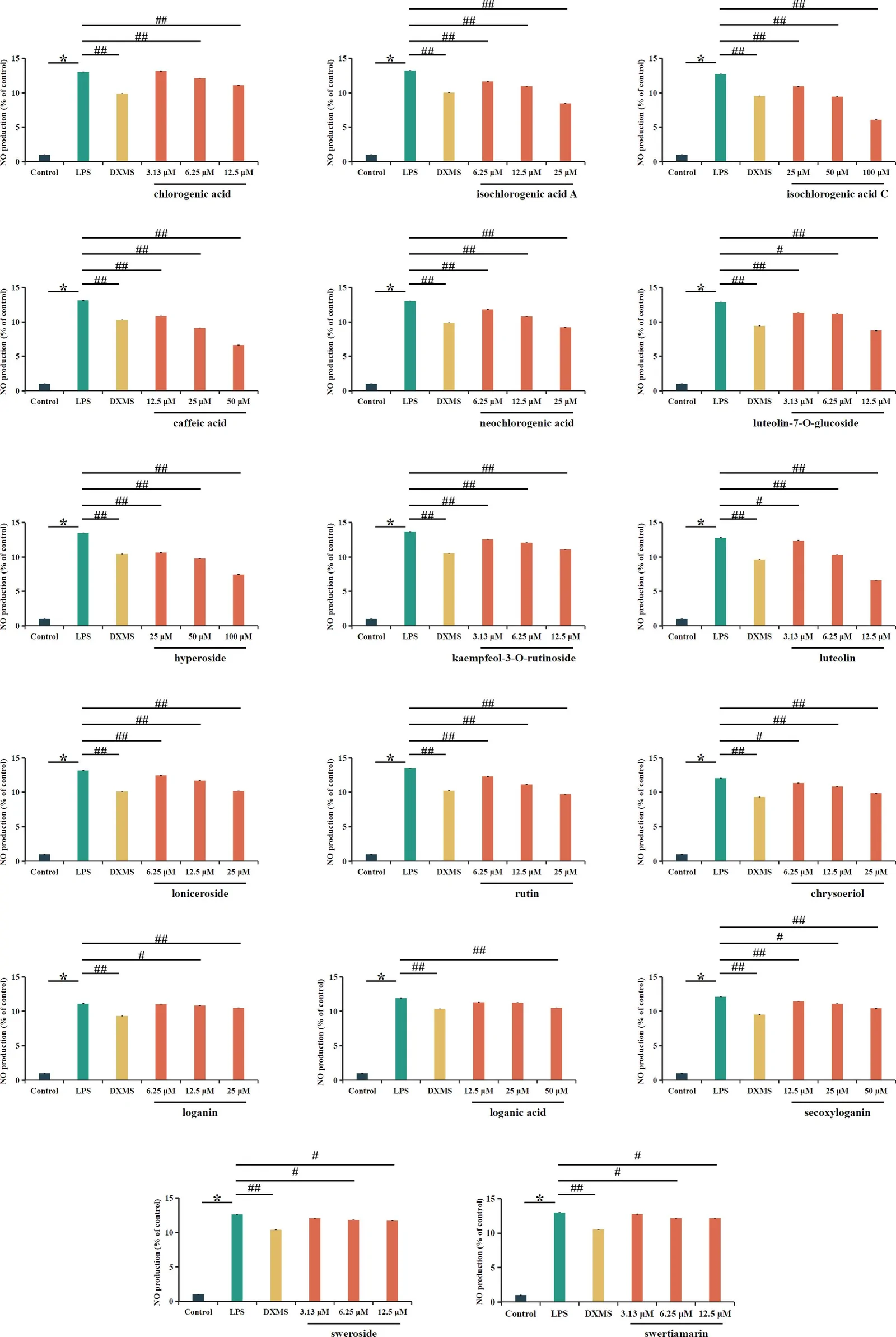
Influence of candidate chemical indicators on NO production in LPS-induced RAW264.7 cells. *: *vs* control; *: significant (*P* < 0.05), **: extremely significant (*P* < 0.01). #: *vs* control; #: significant (P < 0.05), ##: extremely significant (P < 0.01).

After uniformization process, NO production results of candidate chemical indicators were obtained and displayed in Fig. 7. Compared with control group, NO production in model group was significantly increased, indicating that the cell model was created successfully. Moreover, compared to model group, *P* values in multiple groups covered chlorogenic acid (6.25 μmol/L, 12.5 μmol/L), isochlorogenic acid A (6.25 μmol/L, 12.5 μmol/L, 25.0 μmol/L), isochlorogenic acid C (25 μmol/L, 50 μmol/L, 100 μmol/L), caffeic acid (12.5 μmol/L, 25.0 μmol/L, 50.0 μmol/L), neochlorogenic acid (6.25 μmol/L, 12.5 μmol/L, 25.0 μmol/L), luteolin-7-O-glucoside (3.13 μmol/L, 6.25 μmol/L, 12.5 μmol/L), hyperin (25.0 μmol/L, 50.0 μmol/L, 100 μmol/L), kaempfeol-3-O-rutinoside (3.13 μmol/L, 6.25 μmol/L, 12.5 μmol/L), luteolin (3.13 μmol/L, 6.25 μmol/L, 12.5 μmol/L), loniceroside (6.25 μmol/L, 12.5 μmol/L, 25.0 μmol/L), rutin (6.25 μmol/L, 12.5 μmol/L, 25.0 μmol/L), chrysoeriol (6.25 μmol/L, 12.5 μmol/L, 25.0 μmol/L), loganic acid (50.0 μmol/L), secoxyloganin (25.0 μmol/L), sweroside (3.13 μmol/L, 6.25 μmol/L, 12.5 μmol/L), and swertiamarin (6.25 μmol/L, 12.5 μmol/L) were less than 0.01. The results explained that these compounds with the above-mentioned concentrations revealed extremely significant inhibition of NO production. In addition, compared to model group, P values in loganin (25.0 μmol/L) group and secoxyloganin (50.0 μmol/L) group were less than 0.05, illustrating that 25.0 μmol/L of loganin and 50.0 μmol/L of secoxyloganin inhibited NO production significantly. In brief, all the above-mentioned candidate chemical indicators exhibited favourable anti-inflammatory activity, and the effect showed dose-dependent characteristic during the specified concentration ranges.

Comprehensively considering the constituents absorbed into blood, transformation processes and classic anti-inflammation activity evaluation, 17 of candidate chemical indicators were finally deemed as the formal chemical indicators, which were practically adopted in the quality control of *Lonicera japonica* from various origins.

### 3.4 Quality control of *Lonicera japonica* from different origins

#### 3.4.1 The linear, precision, repeatability, and stability results

All samples were detected under NEG ion mode in view of the favourable response values of the targeted chemical indicators. The precursor ions and product ions were screened out beforehand based on the data of individual standard. The parameters of MRM under NEG ion mode were displayed in Table 5. In order to detect the targeted chemical indicators with pleased separation simultaneously, the eluent gradient for MRM analysis was as well as optimized. Under the established method, the results of linear, precision, repeatability, stability were displayed in Table 6. All the linear regressions of targeted chemical indicators were larger than 0.99, which showed well fitting in the given concentration ranges. The relative standard deviation (RSD) of precision, repeatability and stability were less than 5%, explaining that the established method was befitting for the determination of targeted chemical indicators. In addition, the detailed information of MS, MS/MS, DP and CE of the selected chemical indicators were displayed in the Supplemental profile.

**Table 5:** The MRM parameters of chemical indicators *via* UHPLC-HRMS tool under NEG ion mode.

No.	Name	Precursor ion(Da)	Product ion(Da)	DP(V)	CE(V)	Retention time(min)
1	chlorogenic acid	353.09	191.06	− 290	− 28	4.3
2	isochlorogenic acid A	515.12	191.05	− 226	− 45	7.2
3	isochlorogenic acid C	515.12	353.09	− 220	− 25	8.4
4	caffeic acid	179.03	135.04	− 160	− 22	4.6
5	neochlorogenic acid	353.09	191.06	− 265	− 29	3.8
6	luteolin-7-O-glucoside	447.09	285.04	− 290	− 37	6.3
7	hyperin	463.09	300.03	− 266	− 37	6.1
8	kaempfeol-3-O-rutinoside	593.15	285.04	− 290	− 43	6.9
9	luteolin	285.04	133.03	− 243	− 46	9.6
10	loniceroside	593.15	285.04	− 290	− 58	6.3
11	rutin	609.15	300.03	− 280	− 51	5.8
12	chrysoeriol	299.06	284.03	− 207	− 28	9.5
13	loganin	435.15	227.09	− 137	− 11	4.7
14	loganic acid	375.13	213.08	− 180	− 23	3.9
15	secoxyloganin	403.12	371.10	− 157	− 19	5.1
16	sweroside	403.12	125.02	− 157	− 28	4.8
17	swertiamarin	419.12	179.06	− 142	− 17	4.4

**Table 6:** The linear, precision, repeatability, stability of the chemical indicators by UHPLC-HRMS under MRM mode.

No.	Name	Linear			Precision	Repeatability	Stability
		Equation	r	Range (ng/mL)	RSD (%)	RSD (%)	RSD (%)
1	chlorogenic acid	Y = 4.95649× + 104.80785	0.9921	0.605–9.92 × 10^3^	4.6	4.6	3.5
2	isochlorogenic acid A	Y = 9.09829× + 17.17030	0.9943	3.63–5.95 × 10^4^	3.4	4.4	2.5
3	isochlorogenic acid C	Y = 32.94124× − 568.35550	0.9978	1.14–1.86 × 10^4^	4.1	3.1	4.5
4	caffeic acid	Y = 72.64782× + 79.64941	0.9942	0.249–4.08 × 10^3^	4.6	4.3	4.7
5	neochlorogenic acid	Y = 4.64669× − 19,541.80136	0.9926	6.93–1.14 × 10^5^	4.9	3.6	4.1
6	luteolin-7-O-glucoside	Y = 88.41364× − 47.37022	0.9954	0.487–7.98 × 10^3^	3.6	2.7	3.3
7	hyperin	Y = 24.17796× − 739.93607	0.9903	1.25–2.05 × 10^4^	4.0	4.7	2.4
8	kaempfeol-3-O-rutinoside	Y = 46.43463× − 1440.04112	0.9922	1.07–1.75 × 10^4^	3.2	4.6	4.6
9	luteolin	Y = 88.41364× − 47.37022	0.9954	0.487–7.98 × 10^3^	3.9	4.7	3.4
10	loniceroside	Y = 54.80782× −1609.74510	0.9991	1.03–1.68 × 10^4^	3.7	3.8	2.9
11	rutin	Y = 35.15883× − 2245.71831	0.9951	1.23–2.01 × 10^4^	3.5	3.4	4.8
12	chrysoeriol	Y = 145.19975× + 156.31030	0.9965	0.089–1.46 × 10^3^	4.4	3.6	4.7
13	loganin	Y = 4.34211× + 65.62747	0.9979	0.123–2.01 × 10^3^	4.1	4.5	4.3
14	loganic acid	Y = 10.32334× − 341.92531	0.9933	1.32–2.17 × 10^4^	4.4	3.7	4.3
15	secoxyloganin	Y = 7.94888× − 239.91472	0.9993	1.25–2.05 × 10^4^	4.1	4.3	4.4
16	sweroside	Y = 3.25431× − 7.42660	0.9996	4.94–8.10 × 10^4^	4.7	3.9	4.2
17	swertiamarin	Y = 12.94821× − 47.06116	0.9954	1.23–2.02 × 10^4^	4.1	4.2	4.8

#### 3.4.2 Results of content comparison and correlation analysis

**Fig. 8:**
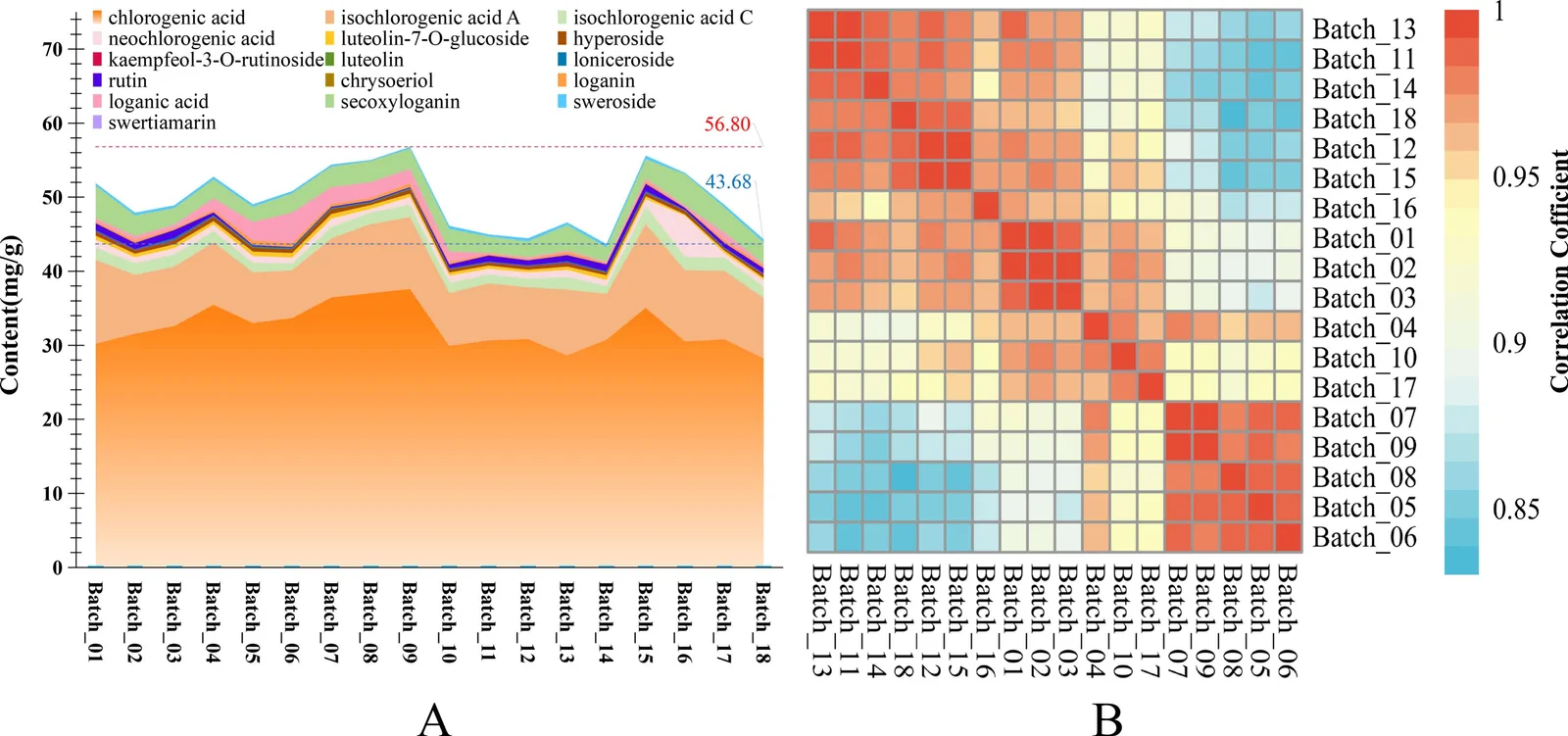
The accumulated contents and correlation analysis of chemical indicators in *Lonicera japonica* from different batches. A: The accumulated contents of chemical indicators in *Lonicera japonica* from different batches. B: The correlation coefficient analysis of chemical indicators in *Lonicera japonica* from different batches. Batch 1–6 were from Shandong Province in China, Batch 7–12 were from Henan Province in China, Batch 13–18 were from Hebei Province in China.

Before the determination of chemical indicators in *Lonicera japonica* from different origins, water contents were detected by toluene method (Xiang, Ouyang, Li, & Fu, 2021). Then, based on the optimized MRM conditions, the contents of chemical indicators in *Lonicera japonica* from 18 batches were determined concurrently. It was worth noting that all the selected chemical indicators exhibited anti-inflammatory activity, which was in accordance with the classic clinical application of *Lonicera japonica* (Jeong et al., 2023). In view of the common biological activity, the screened multiple chemical indicators in *Lonicera japonica* may perform similar function *in vivo* synergistically. Therefore, compared to simple individual content, accumulated content is more scientific to evaluate the quality of *Lonicera japonica* from diverse origins. Besides, correlation analysis, a method reflecting the similarity of quality (Bing et al., 2025; Li et al., 2025), was as well as used for the quality control. The accumulated contents and correlation results of *Lonicera japonica* from 18 batches were exhibited in Fig. 8. The accumulated contents were ranged from 43.68 mg/g to 56.80 mg/g. The correlation coefficient was higher than 0.85, indicating that *Lonicera japonica* from different origins exhibited high similarity. These results manifested that the screened chemical indicators could comprehensively reveal the quality of *Lonicera japonica* from various batches. Nevertheless, in the present study, due to the limitation of planting origins, there were only 18 batches of *Lonicera japonica* collected. In the future, as the planting areas increases, *Lonicera japonica* from more origins could be assessed by the screened multi-indicators.

## 4 Conclusion

This study offered a comprehensive strategy for multivariate quality description of *Lonicera japonica* based on UHPLC-HRMS approach *via* diverse data acquisitions. Totally 115 of chemical profiles in the gradient-solvent extract of *Lonicera japonica* were identified *via* IDA mode. Abundant bio-active substances covered flavonoids, prenol lipids, alcohols and polyols were extracted simultaneously through coincident technology. In view of the overall analysis *in vitro* and *in vivo*, 17 of ingredients were confirmed as the chemical indicators of *Lonicera japonica*. Based on synchronous quantitative determination *via* MRM mode, the chemical indicators were used for evaluating the overall quality of *Lonicera japonica* scientifically. Our study improved the quality control scheme of *Lonicera japonica*, which provided a novel paradigm for other plant. Moreover, the adopted integrating UHPLC-HRMS tool showed high-throughput and ultra-efficient characteristics, which could be developed as a one-stop analysis platform for chemical study.

## CRediT authorship contribution statement

**Yumei Wang:** Writing – review & editing, Writing – original draft, Visualization, Validation, Supervision, Software, Resources, Project administration, Methodology, Investigation, Funding acquisition, Formal analysis, Data curation, Conceptualization. **Meiling Gu:** Writing – review & editing, Writing – original draft, Visualization, Validation, Supervision, Software, Investigation, Formal analysis, Data curation. **Honglian Zhang:** Supervision, Resources, Methodology, Investigation. **Yujian Han:** Visualization, Validation, Supervision, Data curation. **Ruoxin Li:** Validation, Supervision, Investigation, Funding acquisition. **Yanjie Dai:** Validation, Supervision, Investigation. **Yabin Zhao:** Validation, Supervision, Investigation. **Qi Liu:** Writing – review & editing, Writing – original draft, Visualization, Validation, Supervision, Software, Resources, Project administration, Methodology, Investigation, Funding acquisition, Formal analysis, Data curation, Conceptualization.

## Ethics declaration

This study was conducted in accordance with the ARRIVE (Animal Research: Reporting of In Vivo Experiments) guidelines. This study was approved by the Qiqihar Medical University. (Approval No. QMU-AECC-2021-217)

## Declaration of competing interest

The authors declare that they have no known competing financial interests or personal relationships that could have appeared to influence the work reported in this paper.

## Data Availability

Data will be made available on request.
